# Safety, Tolerability, and Pharmacokinetics of NTM-1632, a Novel Mixture of Three Monoclonal Antibodies against Botulinum Toxin B

**DOI:** 10.1128/AAC.02329-20

**Published:** 2021-06-17

**Authors:** J. T. Guptill, S. M. Raja, V. C. Juel, E. B. Walter, M. Cohen-Wolkowiez, H. Hill, E. Sendra, B. Hauser, P. Jackson, G. K. Swamy

**Affiliations:** a Department of Neurology, Duke University, Durham, North Carolina, USA; b Duke Clinical Research Institute, Durham, North Carolina, USA; c Duke Early Phase Clinical Research Unit, Durham, North Carolina, USA; d Department of Pediatrics, Duke University School of Medicine, Durham, North Carolina, USA; e Duke Human Vaccine Institute, Duke University School of Medicine, Durham, North Carolina, USA; f Department of Obstetrics and Gynecology, Duke University School of Medicine, Durham, North Carolina, USA; g The EMMES Corporation, Rockville, Maryland, USA

**Keywords:** *Clostridium botulinum*, clinical trials, monoclonal antibodies

## Abstract

Botulism is a rare, life-threatening paralytic disease caused by Clostridium botulinum neurotoxin (BoNT). Available treatments, including an equine antitoxin and human immune globulin, are given postexposure and challenging to produce and administer. NTM-1632 is an equimolar mixture of 3 human IgG monoclonal antibodies, B1, B2, and B3, targeting BoNT serotype B (BoNT/B). This first-in-human study assessed the safety, tolerability, pharmacokinetics (PK), and immunogenicity of NTM-1632. This double-blind, single-center, placebo-controlled dose escalation study randomized 3 cohorts of healthy volunteers to receive a single intravenous dose of NTM-1632 (0.033, 0.165, or 0.330 mg/kg) or saline placebo. Safety monitoring included physical examinations, clinical laboratory studies, and vital signs. Blood sampling was performed at prespecified time points for PK and immunogenicity analyses. Twenty-four subjects received study product (18 NTM-1632; 6 placebo), and no deaths or serious adverse events were reported. Adverse events in the NTM-1632 groups were generally mild and similar in frequency and severity to the placebo group, and no safety signal was identified. NTM-1632 has a favorable PK profile with a half-life of >20 days for the 0.330-mg/kg dose and an approximately linear relationship with respect to maximum concentration and area under the concentration-time curve (AUC_0→_*_t_*). NTM-1632 demonstrated low immunogenicity with only a few treatment-emergent antidrug antibody responses in the low and middle dosing groups and none at the highest dose. NTM-1632 is well tolerated at the administered doses. The favorable safety, PK, and immunogenicity profile of NTM-1632 supports further clinical development as a treatment for BoNT/B intoxication and postexposure prophylaxis. (This study has been registered at ClinicalTrials.gov under identifier NCT02779140.)

Botulinum toxin (BoNT), the most potent toxin known to humans (1, 2), is produced by the obligate anaerobic organism Clostridium botulinum, with 7 neurotoxin serotypes identified to date (A to G) (3, 4). Mechanisms of BoNT intoxication include ingestion of food contaminated by preformed toxin, infection of wounds (5), toxicoinfection in infants and adults, and iatrogenic chemodenervation (6). An additional theoretical mechanism is dispersal of a weaponized toxin (7, 8). After toxicoinfection in infants, foodborne intoxication is most common; there is also a rising incidence of wound botulism among intravenous drug users (9). Serotypes A (48%), B (49%), and E (2%) accounted for 181 of the 182 cases of confirmed botulism reported to the Centers for Disease Control and Prevention (CDC) in 2017 (9). Typical clinical symptoms include weakness, autonomic symptoms, and symmetric descending paralysis beginning with the cranial nerves (10). Exposure to BoNT can result in respiratory failure within days to weeks and sporadic cases were associated with over 60% mortality prior to the 1950s; the availability of modern supportive care (11) and the development of antitoxins (12) have reduced the overall mortality to 3% (range, 0.8 to 34.4%) (13–15).

Current therapies for botulism are largely given postexposure when an individual is symptomatic. Presently, the only postexposure therapies available include an equine antitoxin (investigational heptavalent botulinum antitoxin [HBAT]) (16) and a human immune globulin reserved for infant use (botulism immune globulin intravenous [BIG-IV]) (17). Production of both the antitoxin and immune globulin is labor-intensive and costly, limiting large-scale manufacture. Furthermore, equine antitoxin preparations are immunogenic due to remnant antibody fragments (Fc region) and associated with a high incidence of acute or delayed hypersensitivity reactions (18). The equine antitoxin is also known to have components with a short serum half-life that increases potential for reintoxication following initial infusion therapy (19). Human immune globulin production is also complicated by the potential for variable antibody composition, potency, dosing, and safety profile between individual product lots.

Given the challenges of antitoxin and immune globulin production and administration, there is a strong unmet need for novel therapeutics to treat patients with botulism and have a scalable therapeutic for postexposure prophylaxis. Through support of the National Institute of Allergy and Infectious Diseases (NIAID), Ology Biosciences, Inc., is developing comixtures of human monoclonal IgG_1_ antibodies against BoNT serotypes A (BoNT/A, NTM-1631 [formerly XOMA 3A]) and B (BoNT/B, NTM-1632 [formerly XOMA 3B]) for prevention and treatment of botulism (20). Comixture of human monoclonal antibodies against BoNT serotypes C (BoNT/C) and D (BoNT/D) have also been developed for prevention and treatment of botulism (21). NTM-1632 is an equimolar mixture of 3 IgG monoclonal antibodies, B1, B2, and B3; each antibody consists of a distinct light- and heavy-chain variable region that binds to a nonoverlapping epitope on BoNT serotype B (BoNT/B) and common human gamma-1 and kappa constant regions. As NTM-1632 is structurally similar to NTM-1631, the mechanism of action is likely similar: high-affinity binding of the mixture to toxin, blockade of cellular binding epitopes on the toxin, and increased hepatic clearance of the toxin-Ab immune complexes (20, 22, 23). Based on the promising preclinical testing (24, 25) and the positive results from the phase I trial of NTM-1631 (20, 26), the human investigational product NTM-1632 is a promising agent for the treatment and prophylaxis of botulism caused by BoNT/B. The purpose of this study was to assess the safety, tolerability, pharmacokinetics (PK), and immunogenicity of intravenously administered escalating single doses of NTM-1632.

## RESULTS

### Demographic characteristics.

A total of 24 subjects were randomized and received NTM-1632 or placebo. All subjects received full study treatment and completed follow-up. Within each cohort (*n* = 8), 6 subjects received NTM-1632 (cohort A, 0.033 mg/kg of body weight; cohort B, 0.165 mg/kg; or cohort C, 0.33 mg/kg) and 2 subjects received placebo.

Subjects ranged in age from 22 years to 45 years; body mass index (BMI) ranged from 21.1 to 30 kg/m^2^. Subjects were predominantly white (13 [54%]) and non-Hispanic or Latino (23 [96%]); the proportion of males was equal to females in all cohorts except the placebo group, in which 4 subjects were female. The demographic characteristics of the individual cohorts are summarized in Table 1.

**TABLE 1:** Demographics of the study population

Characteristic	Values for subjects receiving NTM-1632 (mg/kg) at^*a*^:				
	0.033 (*n* = 6)	0.165 (*n* = 6)	0.330 (*n* = 6)	Placebo (*n* = 6)	All subjects (*n* = 24)
Age					
Mean (SD)	26.2 (3.7)	29.8 (5.7)	30.5 (7.9)	33.8 (6.5)	30.1 (6.4)
Median	26.0	28.5	30.0	31.5	29.0
Min, max^*b*^	22, 32	24, 40	23, 45	28, 45	22, 45
BMI^*c*^ (kg/m^2^)					
Mean (SD)	27.2 (2.4)	27.3 (3.1)	24.1 (3.0)	25.5 (3)	26.0 (3.1)
Median	26.5	28.8	23.9	25.5	25.6
Min, max	24.6, 30.2	22.4, 30.1	21.1, 29.0	21.0, 30.5	21.0, 30.5
Sex					
Male	3 (50)	3 (50)	3 (50)	2 (33)	11 (46)
Female	3 (50)	3 (50)	3 (50)	4 (67)	13 (54)
Race					
White	2 (33)	1 (17)	6 (100)	4 (67)	13 (54)
Black or African-American	4 (67)	4 (66)	0 (0)	2 (33)	10 (42)
Native Hawaiian or other Pacific Islander	0	1 (17)	0 (0)	0 (0)	1 (4)
Ethnicity					
Hispanic or Latino	1 (17)	0 (0)	0 (0)	0 (0)	1 (4)
Non-Hispanic or Latino	5 (83)	6 (100)	6 (100)	6 (100)	23 (96)

a Data are expressed as number (%) unless stated otherwise.

b Min, minimum; max, maximum.

c BMI, body mass index.

### Safety profile.

No deaths or serious adverse events (SAEs) were reported. A total of 39 AEs (34 mild and 5 moderate severity) were reported for 19 (79%) enrolled subjects: 5 (83%) subjects in the 0.033-mg/kg NTM-1632 group, 4 (67%) in the 0.0165-mg/kg NTM-1632 group, 4 (67%) in the 0.330-mg/kg NTM-1632 group, and 6 (100%) in the placebo group. Of the reported events, 3 (8%) were deemed treatment emergent and related. The related events were mild in severity and resolved within 1 day of onset; two (“throbbing pressure on forehead” and “lightheaded”) were reported in the 0.033-mg/kg NTM-1632 group, and one (“loose stools”) was reported in the 0.33-mg/kg NTM-1632 group. A total of 5 (13%) unrelated, moderate-severity AEs were reported: 3 by placebo subjects, 1 by a 0.033-mg/kg NTM-1632 subject, and 1 by a 0.330-mg/kg NTM-1632 subject.

AEs reported by 2 or more subjects included sinus bradycardia (42%), upper respiratory tract infection (25%), headaches (17%), and myalgia (8%). Of the subjects reporting headaches, 3 (100%) received placebo, and of the subjects reporting upper respiratory tract infection, 2 (33%) received 0.330 mg/kg NTM-1632. One subject reporting myalgia received 0.330 mg/kg NTM-1632 and one received placebo. No other Medical Dictionary for Regulatory Activities (MedDRA)-preferred term was reported by more than one subject in a group. A breakdown of all AEs occurring in at least 10% of subjects are shown by MedDRA-preferred term in Table 2.

**TABLE 2:** Summary of adverse events by subject with an overall rate of ≥10%

Adverse event	Values [no. (%)] for subjects receiving NTM-1632 at (mg/kg):					
	0.033 (*n* = 6)	0.165 (*n* = 6)	0.330 (*n* = 6)	Combined (*n* = 18)	Placebo (*n* = 6)	All subjects (*n* = 24)
Sinus bradycardia	1 (16.7)	2 (33.3)	3 (50.0)	6 (33.3)	4 (66.7)	10 (42)
Upper respiratory tract infection	1 (16.7)	1 (16.7)	2 (33.3)	4 (22.2)	2 (33.3)	6 (25.0)
Headache	1 (16.7)	0 (0)	0 (0)	1 (5.6)	3^*a*^ (50.0)	4 (16.7)
Creatine kinase elevated	2 (33.3)	0 (0)	1 (16.7)	3 (16.7)	1 (16.7)	4 (16.7)
Aldolase increased	1 (16.7)	2 (33.3)	1 (16.7)	4 (22.2)	0 (0)	4 (16.7)
Indirect bilirubin increased	2 (33.3)	1 (16.7)	1 (16.7)	4 (22.2)	0 (0)	4 (16.7)
Hemoglobin decreased	2 (33.3)	1 (16.7)	3 (50.0)	6 (33.3)	1 (16.7)	7 (29.2)
Neutropenia	1 (16.7)	0 (0)	2 (33.3)	3 (16.7)	1 (16.7)	4 (16.7)

a Four headaches in total were reported by 3 subjects in the placebo group.

### Laboratory analyses.

Safety laboratory studies were performed as described above. A total of 14 related biochemistry results, all mild, were reported in 7 subjects, 6 of whom received NTM-1632. A total of 5 severe laboratory results, all deemed unrelated, were reported for 2 subjects. These severe laboratory AEs involved elevations in creatine kinase and aldolase without clinical symptoms in 2 subjects who reported heavy exercise and received 0.033 mg/kg or 0.330 mg/kg NTM-1632. No notable coagulation results were reported. A total of 25 related hematology results were reported in 7 subjects, 6 of whom received NTM-1632. The abnormalities primarily included reductions in hemoglobin and transient neutropenia in subjects who received NTM-1632 (Table 2). One placebo subject developed a transient leukocytosis. All of the abnormalities were graded as mild (22) or moderate (3). The abnormalities resolved by the final visit in all subjects, except for one 0.033-mg/kg NTM-1632 recipient’s hemoglobin; the investigators considered the subject stable and no additional monitoring was required.

Negative pregnancy tests were required in all females of childbearing potential at screening and admission to the confinement unit, and female subjects were required to practice abstinence or use contraception through day 91. Despite reported adherence to contraceptive use guidance, one 0.033-mg/kg NTM-1632 recipient had a positive pregnancy test approximately 4 weeks following investigational medicinal product (IMP) administration and indicated an intent to terminate the pregnancy. Additional information was unavailable, as the subject failed to respond to multiple telephone calls and a certified letter from the site.

### Pharmacokinetic analysis.

**FIG 1:**
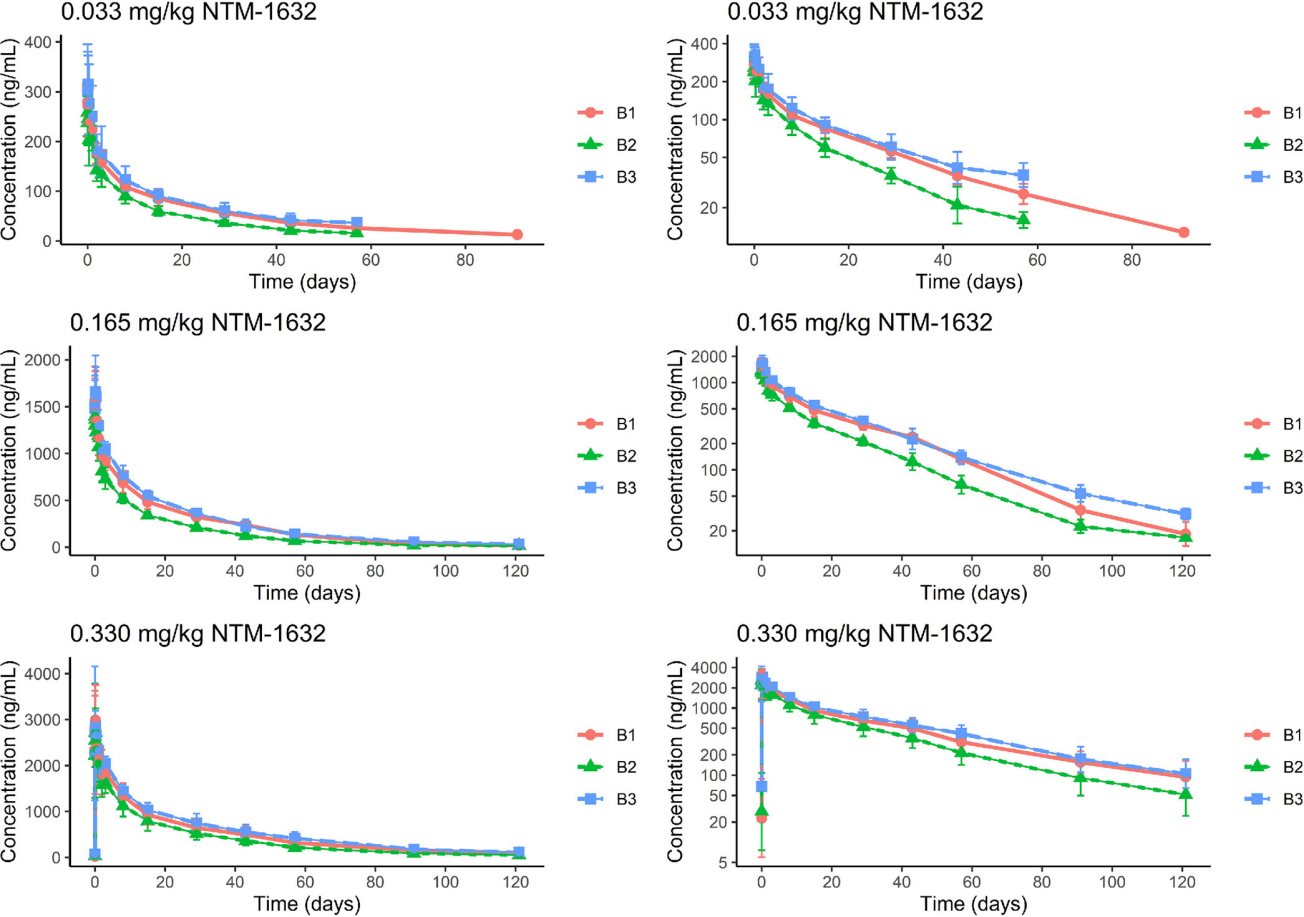
Time courses of serum MAb concentrations after single intravenous (i.v.) dose administration of NTM-1632 are shown on the left, with semilog concentration plots shown on the right. NTM-1632 component MAbs are denoted B1 (red), B2 (green), and B3 (blue). Each row represents separate dosing levels of NTM-1632, with the top-row subjects receiving 0.033 mg/kg, the middle-row subjects receiving 0.165 mg/kg, and the bottom-row subjects receiving 0.330 mg/kg.

A summary of the PK data is presented in Table 3 and Fig. 1. The median maximum observed concentration (*C*_max_) achieved following a single intravenous dose of 0.033 mg/kg, 0.165 mg/kg, and 0.330 mg/kg NTM-1632 was similar for B1, B2, and B3. Median time to maximum concentration (*t*_max_) was achieved in 1.02 to 2.5 h for B1 and B2 in all dose groups. The median *t*_max_ was mildly prolonged for B3 (2.99 to 4.01 h). The median elimination half-life (*t*_1/2_) was 18.8 to 26.8 days in the 0.033-mg/kg and 0.165-mg/kg dose groups (*t*_1/2_ could not be calculated for B2 and B3 lowest doses) and increased slightly to 24.9 to 31.8 days in the 0.330-mg/kg dose. The areas under the concentration-time curve from 0 to *t* (AUC_0-_*_t_*; ng × h/ml) were similar for B1, B2, and B3 in all dose groups, although B2 AUC exposures were lower than B1 and B3 exposures at all doses. Dose proportionality curves for *C*_max_ and AUC_0-∞_ revealed an approximately linear relationship with respect to both *C*_max_ and total AUC exposure, suggesting that all components of NTM-1632 exhibit linear kinetics. Clearance was similar across all component monoclonal antibodies (MAbs) and doses, ranging from 0.189 ml/h/kg to 0.385 ml/h/kg. Apparent volume of distribution at steady state (*V*_ss_) was consistent, ranging from 169 to 236 ml/kg.

**TABLE 3:** Summary of pharmacokinetic parameters for NTM-1632^*d*^

MAb	Cohort^*a*^	*C*_max_ (ng/ml)	*t*_max_ (h)	*t*_1/2_ (days)	AUC_0-_*_t_* (ng × h/ml)	CL^*c*^ (ml/h/kg)	V_ss_ (ml/kg)
B1	A	272 (248, 352)	1.02 (1, 4)	26.8 (26.5, 26.8)	105,000 (65,400, 114,000)	0.27 (0.262, 0.27)	236 (232, 250)
	B	1,650 (1,370, 2,100)	1.21 (1.02, 4)	21.6 (17.1, 24.5)	615,000 (571,000, 746,000)	0.262 (0.218, 0.283)	187 (139, 223)
	C	3,270 (1,940, 3,270)	1.99 (1, 4.05)	31.8 (24.6, 37.2)	1,340,000 (994,000, 1,730,000)	0.234 (0.172, 0.291)	212 (185, 347)
B2	A	257 (192, 341)	1.05 (1, 4)	—^*b*^	66,400 (55,100, 821,000)	—^*b*^	—^*b*^
	B	1,470 (1,310, 1,660)	2.5 (1.05, 4.13)	18.8 (15.2, 23.3)	416,000 (358,000, 472,000)	0.385 (0.349, 0.426)	222 (199, 269)
	C	2,830 (1,910, 3,660)	1.04 (1, 1.04)	24.9 (21.2, 33.6)	1,040,000 (750,000, 1,560,000)	0.314 (0.188, 0.432)	236 (188, 315)
B3	A	330 (257, 450)	3.98 (1.02, 8)	—^*b*^	91,900 (52,900, 132,000)	—^*b*^	—^*b*^
	B	1,580 (1,390, 2,160)	4.01 (1.05, 8.08)	24 (17.3, 26)	690,000 (545,000, 769,000)	0.229 (0.207, 0.27)	169 (134, 199)
	C	2,900 (2,710, 3,530)	2.99 (1, 8)	31 (25.8, 35.3)	1,610,000 (1,200,000, 1,910,000)	0.189 (0.155, 0.264)	202 (177, 213)

a Dosing by cohort: A, 0.033 mg/kg; B, 0.165 mg/kg; C, 0.330 mg/kg.

b Values not estimable.

c CL, clearance.

d Median values with (min, max) are reported.

### Immunogenicity analysis.

Immunogenicity analysis was performed with detection of antidrug antibody (ADA) as described above. Four subjects had preexisting ADA to MAb components of NTM-1632 (1 B1, 2 B2, and 1 B3). A single placebo subject had a preexisting ADA response to B1, and a single placebo subject developed a treatment-emergent response to B2. Treatment-emergent ADA responses were observed in 1 B2 subject (0.165-mg/kg dose) and 2 B3 subjects (1 each at the 0.033-mg/kg and 0.165-mg/kg doses). The B2 treatment-emergent ADA response was a low titer (10) measurable on day 29 and day 57 only. The B3 treatment-emergent ADA responses were also a low titer (10 to 180) measurable at day 15, day 29, day 57, and day 91 in the low-dose group and at day 29 in the middle-dose group. No subjects developed ADA in the highest dosing group. There were no treatment-boosted ADA responses.

## DISCUSSION

This study was a first-in-human assessment of NTM-1632, a BoNT/B product, in healthy adult subjects. Single escalating doses of NTM-1632 administered intravenously to participants were well tolerated over the investigational dose range. AEs in the NTM-1632 cohorts were generally mild and similar in frequency to the subjects receiving placebo. There were no SAEs, and no specific safety signal was identified in this study. In contrast to experience with equine antitoxins, no serious infusion or hypersensitivity reactions occurred. Antibody levels were detectable up to 121 days in the 0.165-mg/kg and 0.330-mg/kg dose groups, and the estimated half-lives of the individual components B1, B2, and B3 were 32, 25, and 31 days, respectively, for the highest dosing group (0.330 mg/kg). This longevity offers a marked advantage over the current HBAT antitoxin with its documented half-life of approximately 7 h for the most rapidly degraded component, serotype E (19). NTM-1632 has a favorable immunogenicity profile. A total of three participants developed transient, low-titer antibodies to the constituent components of NTM-1632 following dosing, and there were no treatment-boosted ADA responses. The highest dose tested, 0.330 mg/kg, is well tolerated, achieved the highest blood concentrations with the longest half-life, and did not cause demonstrable immunogenicity. Based on the completed mouse protection assay and extrapolation to humans, a 0.330-mg/kg dose would be expected to neutralize circulating BoNT/B at the levels tested in preclinical models (27).

In 2002, the National Institute of Allergy and Infectious Diseases identified BoNTs as a category A biothreat and prioritized development of new antitoxins, vaccines, and diagnostic tests (28). Likewise, the Federal Experts Security Advisory Panel, created in response to Executive Order 13546, classified BoNTs as tier 1 agents, highlighting their importance as a national security threat (29). Epidemiologic modeling studies in North America performed to estimate the impact of a large-scale release of BoNT revealed startling numbers: 30,000 fatalities and estimated cost of over $8 billion following exposure of 100,000 individuals (30). Additional estimates predict 1.5 million fatalities from the release of as little as 1 g aerosolized BoNT in a heavily populated area (31). The devastating potential of weaponized BoNTs makes the development of highly efficacious, scalable, and rapidly deployable therapeutics essential (32).

Investment in new therapeutics for botulism has led to the development of human MAbs capable of neutralizing BoNTs. These agents offer several important advantages over existing therapies, particularly the ability to be produced from tissue culture rather than a live host, low risk of hypersensitivity reactions, and longer half-lives that reduce the risk of reintoxication. *In vitro* studies of NTM-1632 demonstrated no specific binding to any of the human tissues evaluated at an optimal concentration of 1.0 μg/ml or high concentration of 5.0 μg/ml (27). This finding provides evidence that NTM-1632 does not bind human epitopes, indicating it is less likely to cause off-target effects and can be administered postexposure without causing adverse effects. Indeed, the first-in-human studies of the BoNT serotype A MAb, NTM-1631, and the BoNT serotype C and D MAb, NTM-1634, revealed the products were well tolerated with a favorable PK profile (20, 21). A novel product combining these comixtures (NTM-1631, NTM-1632, and NTM-1634) has potential to offer broad coverage against multiple serotypes and be rapidly deployed in the event of large outbreaks or weaponization when the serotype is unknown (33). Development of a temperature-stable formulation to neutralize antitoxin BoNT serotypes A, B, and E utilizing this strategy is under way and, to date, demonstrates favorable physiochemical properties (34).

In conclusion, the favorable safety, PK, and immunogenicity profile of NTM-1632 supports further clinical development of this potential treatment for BoNT/B intoxication and postexposure prophylaxis following suspected BoNT/B exposure as part of a broader strategy to develop a new generation of therapeutics targeting botulinum toxin.

## MATERIALS AND METHODS

This is a first-in-human, double-blind, single-center, placebo-controlled dose-escalation study to evaluate the safety and PK of intravenous NTM-1632. Dose selection was based on conversion from the NOAEL dose from GLP studies in Sprague-Dawley rats into a human equivalent dose (HED). Reports on toxicity (NOAEL) are Ology Biosciences licensed. A safety factor of 10 was used to establish a maximal recommended starting dose (MRSD) of 5 mg/kg and further reduced by 152-fold to establish the starting dose of 0.033 mg/kg. A total of three cohorts were planned at doses of 0.033 mg/kg, 0.165 mg/kg, and 0.330 mg/kg. Further dose escalation was deemed unnecessary based on preclinical murine studies demonstrating toxin neutralization in this dose range, which approximates expected human BoNT/B exposure levels.

### Materials.

NTM-1632 is a sterile, clear, colorless, preservative-free, pH 6.0, buffered solution containing a 5-mg/ml equimolar mixture of B1, B2, and B3. The drug was supplied by Ology Biosciences, Inc. Normal saline was used as a placebo.

### Participants.

A total of 24 healthy volunteers and a maximum of 4 alternates per cohort were enrolled. Eligible participants included healthy, nonpregnant, nonlactating female and male subjects aged 18 to 45 years, with BMI between 18.5 and 30 kg/m^2^ and a negative illicit drug and alcohol screen and who agreed to use contraception per protocol. Exclusion criteria included history of recent febrile illness, chronic medical conditions, severe allergic reaction to any type of medication or any study product components, blood donation within 2 months of enrollment, receipt of a blood product within 6 months of enrollment or an antibody within 5 half-lives, treatment with another investigational drug within 1 month of dosing, and use of H1 antihistamines or beta blockers within 5 days of dosing. Additional exclusion criteria included laboratory abnormalities (urinalysis, hematology, and chemistry analysis), positive serology for HIV or hepatitis B or C, and a clinically significant abnormality on electrocardiogram or baseline QT/QTc prolongation.

### Study design.

Research participants were randomized to sequential dose-escalation cohorts consisting of 8 subjects. Within each cohort, participants were randomized in a 3:1 ratio to receive a single intravenous infusion of the IMP or placebo administered over 1 h. Each participant underwent screening followed within 4 weeks by a 3-day inpatient stay at the Duke Early Phase Clinical Research Unit (DEPRU) of Duke University Medical Center. The study was approved by the Duke Health Institutional Review Board, and written informed consent was obtained from all participants prior to screening.

### Study procedures.

Following screening, eligible participants were admitted to the DEPRU 1 day prior to dosing for a complete physical examination, electrocardiogram, and laboratory testing including routine chemistry, hematology, urinalysis, and urine pregnancy test (for females of childbearing potential). Two sentinel subjects in each dosing cohort were randomized 1:1 to receive IMP or placebo prior to dosing the remaining subjects. All study participants who received investigational product were monitored in the DEPRU for 24 h. Subjects in cohort A were followed for 91 days and those in cohorts B and C for 121 days.

### Safety analyses.

Adverse events (AEs) were assessed by targeted interviews for symptoms, physical examination, and laboratory studies from day of infusion through day 57 for all cohorts and classified in accordance with the MedDRA, version 22.0. Relationship to IMP, study procedure, alternate plausible explanation or etiology, severity, and outcome were documented for each AE.

### Laboratory analyses.

Safety laboratory studies (hematology, chemistry, and urinalysis) were conducted on days −1, 2, 4, 8, 15, 29, and 91. Values outside the reference range were recorded as AEs.

### Pharmacokinetics and immunogenicity analyses.

Blood PK samples were drawn prior to infusion and at the end of infusion and at 1, 3, 7, 23, 47, and 71 h following the end of infusion. Additional PK samples were drawn at days 8, 15, 29, 43, 57, 91 (all cohorts), and 121 (cohorts B and C only). Serum samples were analyzed by 3 validated bridging electrochemiluminescence (ECL) assays for the concentrations of each MAb, B1, B2, and B3 (20, 26). Testing was performed by KCAS Bioanalytical & Biomarker Services (Shawnee, KS). The lower limit of quantification for B1, B2, and B3 was 8.00 ng/ml, 10.00 ng/ml, and 25.00 ng/ml, respectively, as expressed in 100% human serum. Concentrations below the lower limit of quantification were set to missing if they occurred after the first positive concentration or imputed to 0 if no positive concentration was ever detected. Missing samples were excluded from PK analysis.

### Immunogenicity.

Serum samples were drawn at days −1, 29, 57, 91 (all cohorts), and 121 (cohorts B and C) for presence of antidrug antibody (ADA) using 3 validated ECL assays, one each for anti-B1, anti-B2, and anti-B3, respectively, for ADA screening, confirmation, and titer measurement. Testing was performed by KCAS Bioanalytical & Biomarker Services (Shawnee, KS). A treatment-emergent response was defined as negative ADA status at baseline and positive ADA status at any point after baseline. A treatment-boosted response was defined as positive ADA status at baseline with an increase in titer in any subsequent posttreatment serum samples that was at least 9-fold over baseline levels.

### Statistical analysis.

Categorical variables were summarized as numbers and percentages and continuous variables as standard deviations (SD), means, and medians. PK parameters included area under the concentration-time curve (AUC), maximum observed concentration (*C*_max_), time to maximum concentration (*t*_max_), elimination rate constant (*k_e_*), terminal half-life (*t*_1/2_), clearance, and volume of distribution (*V*) for each monoclonal component for each dosing cohort. Descriptive statistics were calculated using SAS (v9.4; SAS Institute, Cary, NC), and PK parameters were estimated by noncompartmental methods using WinNonLin (v8.0 or higher; Certara, Princeton, NJ).

### Data availability.

Upon request and in accordance with NIH data sharing agreements and contract requirements, data will be made available as widely as possible while safeguarding the privacy of participants and protecting confidential and proprietary data. Requests should be directed to the corresponding author.
